# Discrimination between G/C Binding Sites by Olivomycin A Is Determined by Kinetics of the Drug-DNA Interaction

**DOI:** 10.3390/ijms21155299

**Published:** 2020-07-26

**Authors:** Artemy D. Beniaminov, Galina V. Chashchina, Mikhail A. Livshits, Olga I. Kechko, Vladimir A. Mitkevich, Olga K. Mamaeva, Anna N. Tevyashova, Alexander A. Shtil, Anna K. Shchyolkina, Dmitry N. Kaluzhny

**Affiliations:** 1 Engelhardt Institute of Molecular Biology, Russian Academy of Sciences, 32 Vavilov Street, 119991 Moscow, Russia; chashchina.g.v@gmail.com (G.V.C.); livshits@eimb.ru (M.A.L.); olga.kechko@gmail.com (O.I.K.); mitkevich@gmail.com (V.A.M.); okm@eimb.ru (O.K.M.); ashchyolkina@gmail.com (A.K.S.); 2 Gause Institute of New Antibiotics, 11 B Pirogovskaya Street, 119021 Moscow, Russia; chulis@mail.ru; 3 Blokhin National Medical Research Center of Oncology, 24 Kashirskoye Shosse, 115478 Moscow, Russia; shtilaa@yahoo.com; 4 Institute of Gene Biology, Russian Academy of Sciences, 34/5 Vavilov Street, 119991 Moscow, Russia

**Keywords:** Olivomycin A, DNA binding, kinetics, sequence specificity

## Abstract

Olivomycin A (OA) exerts its cytotoxic potency due to binding to the minor groove of the G/C-rich DNA and interfering with replication and transcription. Screening of the complete set of tetranucleotide G/C sites by electrophoretic mobility gel shift assay (EMSA) revealed that the sites containing central GC or GG dinucleotides were able to bind OA, whereas the sites with the central CG dinucleotide were not. However, studies of equilibrium OA binding in solution by fluorescence, circular dichroism and isothermal titration calorimetry failed to confirm the sequence preference of OA, indicating instead a similar type of complex and comparable affinity of OA to all G/C binding sites. This discrepancy was resolved by kinetics analysis of the drug–DNA interaction: the dissociation rate significantly differed between SGCS, SGGS and SCGS sites (S stands for G or C), thereby explaining the disintegration of the complexes during EMSA. The functional relevance of the revealed differential kinetics of OA–DNA interaction was demonstrated in an in vitro transcription assay. These findings emphasize the crucial role of kinetics in the mechanism of OA action and provide an important approach to the screening of new drug candidates.

## 1. Introduction

**Figure 1:**
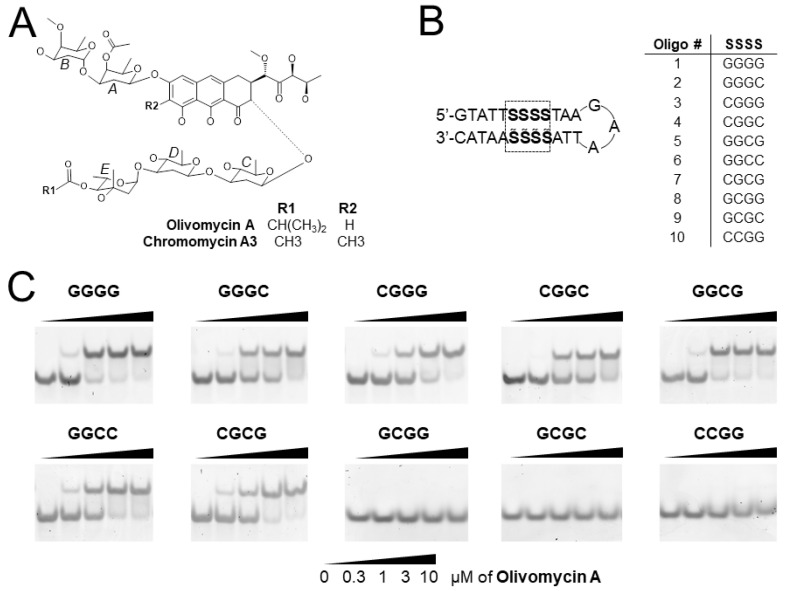
(**A**) Chemical structures of OA and Chro formed by the tricyclic chromophore (aglycon) glycosylated with di- and trisaccharides. (**B**) Ten DNA hairpins covering the full set of tetranucleotide binding sites for OA. S stands for G or C; S with a tilde denotes the nucleotide complementary to S. (**C**) Electrophoretic mobility gel shift assay (EMSA) for the detection of OA binding to ten G/C sites. The concentration of each oligonucleotide in the assay was 50 nM. The gels were stained with SybrGold. Solid wedge represents the increasing concentration of OA.

Antibiotics of the aureolic acid family include structurally close olivomycin A (OA), chromomycin A3 (Chro) and mithramycin (Mith) [1]. Initially, these agents have been considered as antitumor drug candidates due to their potent cytotoxicity for cultured cells [1,2]. Binding of these compounds to GC-rich DNA stretches and, in particular, the prevention of binding of the transcription factors such as SP1 and ETS to DNA provided a mechanistic justification for the therapeutic perspective of the aureolic acid derived agents [3,4,5]. These antibiotics contain an aglycon chromophore bearing A-B disaccharide on one side and the C-D-E trisaccharide on the other side. The chemical structures of OA and Chro are highly similar (Figure 1A); minor differences include methyl groups at position 7 of the aglycon moiety and the acyl residue in sugar E. The antibiotics of this group are able to bind to double-stranded DNA, with a strong preference for G/C rich regions, and are believed to exert their antibacterial and antitumor activities via the inhibition of replication and transcription [6,7], although other targets have been also considered [8]. The molecular basis of preference for G/C rich sequences has been uncovered by numerous studies [9,10,11,12,13]. The antibiotics bind as a divalent metal ion coordinated dimer occupying four consecutive G-C base pairs in the minor groove of the DNA double helix. The X-ray structure of Chro with (TTGGCCAA)_2_ DNA duplex [12] revealed that the metal ion is octahedrally coordinated to O1 and O9 atoms of the chromophore; two water molecules act as the fifth and sixth ligands. The two coordinated water molecules form hydrogen bonds with O2 atoms of cytosine residues, while O8 atoms of the antibiotic make a hydrogen bond to N2 atoms of guanines of the two central GC base pairs in the GGCC binding site. These specific interactions explain the requirement for two central GC base pairs in the binding site. The carbohydrate moieties of the antibiotic (rings A/B and C/D/E) wrap around the minor groove and form an H-bond between the B-sugar of the drug, with phosphate groups of the DNA molecule as well as hydrogen bonds between O1 of the E-sugar ring and N2 of guanines of the extreme GC base pairs in the tetranucleotide binding site. Thus, six GC-specific hydrogen bonds between Chro and DNA provide specificity to the GGCC sequence.

While structural studies have clearly explained the preference for G/C over A/T sequences for these antibiotics, their ability to distinguish between different G/C sequences of the double helix has not been investigated in detail. Partially, this issue was addressed for a subset of self-complementary G/C sequences using several biochemical and biophysical methods that revealed the binding preferences of the Chro dimer in the following order: GGCC > CGCG > CCGG ~ GCGC [14,15]. However, potential binding sites for the antibiotic in the genomic DNA are not restricted to self-complementary sequences. Similarly, Mith differentially interacted with individual G/C tetranucleotide sites, as revealed by DNase and hydroxyl radical probing [16,17,18].

OA is known for its cytotoxicity, antitumor potency and the ability to differentially down-regulate a considerable number of genes. Thus, knowledge of OA’s affinity to any genomic sequence is important for the prediction of preferentially repressed genes as well as for understanding the mechanisms whereby this agent affects transcription or replication. In addition, altered specificity could be the central parameter in developing OA derivatives with milder general toxicity and better tolerance in vivo.

In this study, we dissected the selectivity of OA binding to different G/C rich DNA sequences. To this end, we considered the full set of G/C binding sites and evaluated the ability of each one to accommodate the antibiotic. OA indeed exhibited a nucleotide sequence preference that was shown to stem from the kinetic aspects of the drug–DNA interaction. We demonstrate that this kinetic selectivity might be critical for the mechanism of action of the antibiotic.

## 2. Results

### 2.1. Screening of G/C Tetranucleotide Sites for OA Binding by EMSA

The aureolic family of antibiotics has been known to demonstrate a strong preference for GC-rich regions of the double-stranded DNA. The minimal binding site for OA or structurally close Chro (Figure 1A) spans four consecutive base pairs in the minor groove. Two central nucleotides of the tetranucleotide binding site interact with the aglycon moiety of the antibiotic dimer [12]. To explore the sequence specificity of OA to different GC contexts, we designed a series of oligonucleotides that covered a full set of double-stranded tetranucleotide G/C binding sites. Each oligonucleotide was designed to form a hairpin consisting of a stem and a trinucleotide GAA loop that considerably stabilized the hairpin structure (Figure 1B) [19]. The stem included one single tetranucleotide G/C binding site flanked by several A/T base pairs. The flanking AT sequences identical for all oligonucleotides were designed to avoid the formation of non-hairpin structures. A total ten 27-mers were synthesized and grouped according to the central dinucleotide motif: oligonucleotides 1–4 carried the central GG; nos. 5–7 had GC, whereas nos. 8–10 carried CG dinucleotide (Figure 1B and Table S1).

The ability of oligonucleotides to bind OA was tested in EMSA by differential mobility of free DNA hairpins and the complexes with the drug (Figure 1C). The mobility retardation by OA was observed for oligonucleotides 1–7 at drug concentrations <1 µM, indicating OA–DNA complex formation. The increase of OA concentration to 10 µM led to complete binding of the oligonucleotides. On the contrary, oligonucleotides 8–10 revealed no binding in these assays. Therefore, the hallmark of the oligonucleotides capable of binding OA is the presence of central dinucleotides GG or GC in the tetranucleotide binding site, while the oligonucleotides with the central CG showed no retardation in the gel in the presence of even 10 µM OA.

### 2.2. Equilibrium Binding of OA to G/C Sites

**Figure 2:**
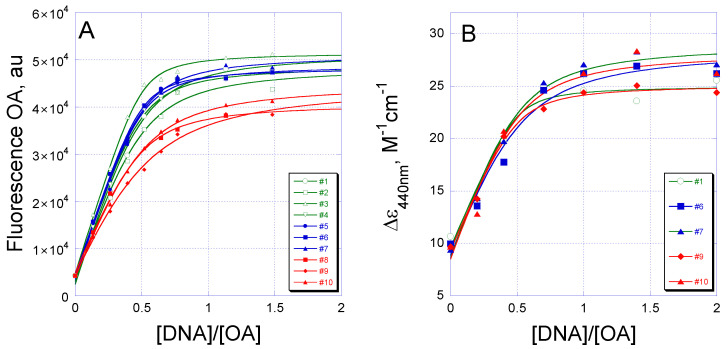
Equilibrium binding of OA to different G/C DNA sites monitored by (**A**) intrinsic fluorescence of bound OA or (**B**) induced CD at 440 nm.

Equilibrium binding of OA to DNA in solution was performed to verify the specificity of OA to the nucleotide context revealed in EMSA. An increase in fluorescence of the aureolic acid family of antibiotics upon binding to DNA provides a quantitative method for the measurement of drug–DNA complex formation. Binding of OA to a DNA hairpin under equilibrium conditions resulted in a 10-fold increase in fluorescence for all 10 tetranucleotide sites (Figure 2A). Similar stoichiometries (2:1 OA:DNA) were observed for all binding sites, indicating no sequence preference for OA. As no dramatic difference between the G/C sites in OA binding was found in the fluorescence assay, we used isothermal titration calorimetry (ITC) for an accurate assessment of the equilibrium binding constant and thermodynamic parameters of OA–DNA interaction.

For ITC measurements, OA was prepared in a magnesium containing buffer to form OA dimers. Then, each DNA hairpin renatured in the same buffer was titrated into the OA solution. This experimental design would ensure the most reliable and accurate measurements since (1) OA dimer would barely dissociate due to minimal dilution of the initial OA solution and (2) stable DNA hairpin would not denature during dilution because its intramolecular structure is stitched by an extremely stable trinucleotide loop structure. The experimental data fitted to the theoretical binding curve in concert with the simple model of a single OA dimer binding to a DNA hairpin (Figure S1). The equilibrium constant, stoichiometry and thermodynamic parameters of OA binding to different DNA sites obtained by ITC are shown in Table 1. The equilibrium association constant turned out to be similar for all G/C binding sites. The maximum difference did not exceed two-fold, which apparently cannot explain the dramatic difference in the behavior of oligonucleotides in EMSA. Both equilibrium constants and stoichiometry argued for a similar mode of interaction between OA and all 10 G/C DNA binding sites.

In the search for structural differences between the complexes formed by OA with G/C DNA binding sites, we applied UV-Vis CD. Representative DNA hairpins 1, 6, 7, 9 and 10 were chosen as they contained central dinucleotides GG, GC and CG in the binding site. Vis-CD spectra of OA (5 µM) mixed with the increasing concentrations of each oligonucleotide resulted in an increase in the 440 nm band. The 440-nm band in the CD spectra demonstrated similar shape and saturation upon titration with oligonucleotides containing central GG, GC and CG (Figure 2B and Figure S2). As the changes in CD spectra around 440 nm reflect the conformational rearrangement of the drug only, these similarities emphasized the similar type of OA dimer structure regardless of the DNA sequence that accommodated the dimer. UV-CD spectra were also monitored, although their interpretation is more complicated since structural alterations in both DNA and OA are responsible for changes in the UV region. However, the UV CD spectra revealed two isosbestic points discernible at ~ 270 nm and 285 nm that implied the concerted structural changes in DNA and OA dimers upon binding (Figure S3). A difference at 260 nm observed for OA-GGGG complexes vs. OA-CGCG or OA-GCGC is probably due to the unique CD spectra of the unbound GGGG hairpin (Figure S4, inset). The band at 260 nm in the differential CD spectra of free hairpins GGGG and GCGC is preserved for the OA–GGGG complex.

A considerable discrepancy between OA specificity obtained by in-gel and in-solution methods can occur for several reasons: (1) a much lower concentration of the drug and DNA in EMSA compared to the equilibrium studies by CD, ITC or intrinsic OA fluorescence, (2) the method of OA–DNA complex visualization in the gel, (3) non-optimal conditions for complex formation in the gel which contains 1 mM EDTA. To examine these possibilities, we performed EMSA at higher concentrations of oligonucleotides (2.5 µM) and OA (5 µM). In these experiments, the gels were visualized in two ways: (1) using intrinsic fluorescence of OA bound to DNA (Figure S5A, upper panel) and (2) after ethidium bromide (EtBr) staining of the same gel, which revealed the bound and free DNA (Figure S5A, lower panel). The shifted electrophoretic mobility of oligonucleotides 1–7 indicated OA–DNA complex formation, whereas the complexes formed by oligonucleotides 8–10 were still absent or weak: the binding sites with the central CG demonstrated little or no interaction in EMSA even after a 50-fold increase in DNA concentration. Millimolar concentrations of EDTA in the gel may cause a substantial bias in the studies of sequence specificity of OA dimers containing the magnesium ion. The EMSA was repeated in the absence of EDTA and in the presence of 1 mM magnesium acetate in the gel (Figure S5B). Qualitatively, the result was not changed in these conditions: the mobility retardation was observed only for hairpins 1–7 but not for hairpins 8–10.

### 2.3. Kinetics of OA Interaction with Different G/C Sites

**Figure 3:**
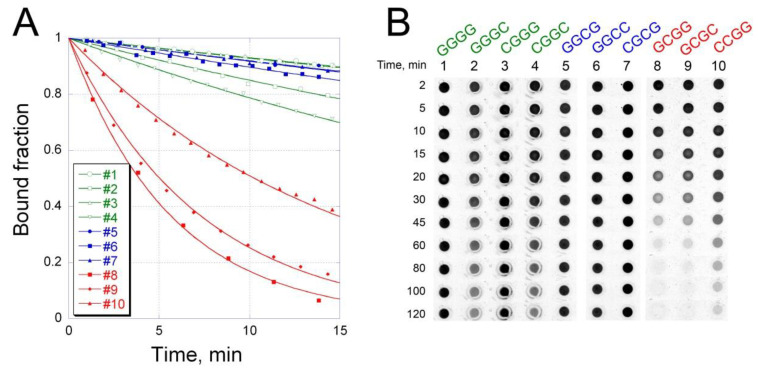
Kinetics of OA dissociation from DNA hairpins carrying 10 tetranucleotide binding sites in solution (**A**) and in 10% polyacrylamide gel (PAAG) (**B**). (**A**) Dissociation of OA–DNA complexes in buffer A was monitored by fluorescence at 550 nm after addition of 1% SDS. (**B**) Spontaneous dissociation of OA–DNA complexes. Complexes of OA with oligonucleotides 1–10 in buffer A were loaded into the wells formed in 10% PAAG. Images were taken under UV at indicated time intervals. Changes in OA fluorescence in the wells reflected time-dependent dissociation of complexes and drug diffusion into the gel. Dissociation curves and loading wells are colored according to the respective binding site: SGGS sites are in green; SGCS in blue; SCGS in red.

To resolve the inconsistency between the results of EMSA and equilibrium studies in solution, we investigated the kinetics of OA–DNA complex formation. A considerable increase in OA fluorescence upon binding to DNA provides an easy approach for monitoring the association/dissociation kinetics of OA–DNA interaction. The rate of complex formation turned out to be independent of the nucleotide sequence of the binding site (Figure S6): the association constants ***k^+^*** calculated for each hairpin were in the range 2–5 × 10^4^ M^−1^ s^−1^ (Table 2). On the contrary, the dissociation rate significantly differed depending on the G/C context of the binding site (Figure 3A). The fastest dissociation was observed for hairpins 8–10, carrying the binding sites with an SCGS pattern. The lifetime of OA complexes with these oligonucleotides was 10–20 min. In striking contrast, OA dissociation from SGCS or SGGS sites was significantly slower (Figure 3A, Table 2).

The lifetimes of OA complexes with different tetranucleotide sites (Table 2) correlated with the fractions of bound OA detectable in EMSA (Figure S5). This observation led us to assume that the differential retardation of DNA hairpins in EMSA was due to the different dissociation rates of the complexes and subsequent passive drug diffusion during electrophoresis. To test this hypothesis, we studied the process of OA dissociation from different DNA binding sites in PAAG in the absence of an electric field. The complexes of OA with DNA hairpins were incubated in buffer A and loaded into the cylindrical wells formed in 10% PAAG. The molecule of the antibiotic, being smaller than the DNA hairpin, would diffuse into the gel more rapidly than DNA. Thus, a gradual decrease in OA fluorescence over time reflects the dissociation of the complex. Figure 3B shows that OA fluorescence initially detectable in each well (2 min after filling the gels) remained unchanged in rows 1, 3 and 5–7 for at least 2 h. In rows 2 and 4, the fluorescence slightly decreased over time. In striking contrast, fluorescence in rows 8–10, containing hairpins with the pattern SCGS, extinguished faster and was barely visible after 2 h.

These results indicate that the central G/C dinucleotide in the tetranucleotide site is critical for the stability of OA–DNA complexes. Indeed, the complexes formed on SGGS or SGCS sites were more stable than those on SCGS sites. Interestingly, the cytosine base adjacent to the central GG dinucleotide at the 3’-end may weaken the stability of OA–DNA complexes, as observed for hairpins 2 (GGGC) or 4 (CGGC) compared to hairpins 1 (GGGG) or 3 (CGGG) (Figure 3B). These in-gel kinetics results are in good agreement with the time course of SDS-induced complex dissociation in solution (Figure 3A and Table 2).

Thus, although ITC showed similar equilibrium binding constants for OA dimers to all ten G/C sites, the antibiotic dissociates from an individual DNA site with a different rate, and this difference fully explains the results of EMSA. In this respect, the similar association kinetics for all ten binding sites, as observed by fluorescence of bound OA, is unexpected and contradictory. The kinetic and equilibrium results can be reconciled assuming that the drug–DNA association is not a one-step process. It is known that, in addition to SSSS sites, Chro, the most structurally close to OA, can still bind to the sites containing one or two A-T base pairs at the extreme positions—that is, (A/T)SSS or SSS(A/T) or (A/T)SS(A/T)—but with a lower affinity [14,15]. Thus, the OA dimer may potentially bind each hairpin in one of three different frames. This initial rapid step in the antibiotic’s accommodation is monitored by fluorescence. At the second step, OA shifts to a more thermodynamically preferred SSSS position. This step is slower and not detectable by fluorescence. The kinetics of the second step may differ for SGGS, SGCS and SCGS sites. The proposed model can explain why the rates of OA binding to different DNA sequences were identical.

### 2.4. Inhibition of T7 Transcription by OA at Different G/C Sites

The cytotoxicity of OA is attributed to the inhibition of transcription and/or DNA replication. Having established the kinetic parameters of discrimination of individual G/C sites by OA, we were interested in whether the observed difference may have functional relevance. We tested OA effects on in vitro transcription by T7 RNA polymerase (RNAP). Three double-stranded DNA templates for T7 RNAP contained a 17-nt T7 promoter and an OA binding site, CGCG, GCGC or GGGG, positioned 15 nucleotides downstream of the transcription start site (Table S1). These templates were used in a run-off transcription; the reaction’s readout was the amount of 31-nt full length RNA product.

**Figure 4:**
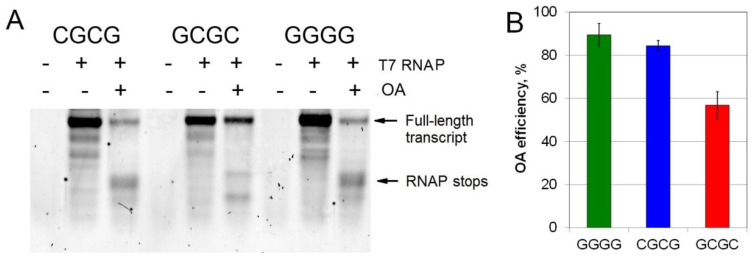
Run-off T7 transcription performed from the short double-stranded DNA templates containing a single tetranucleotide OA binding site, CGCG, GCGC or GGGG. (**A**) Separation of the transcription products in a 10% denaturing polyacrylamide gel. The arrows indicate the full-length RNA transcript and RNAP stop points in the presence of OA. (**B**) The efficiency of transcription inhibition by OA is calculated as percentage of (R_F_ – R_OA_)/R_F_, where R_F_ and R_OA_ are the amount of the full-length RNA product produced in the absence and presence of OA, respectively.

Figure 4 demonstrates that OA impeded transcription at each of the three binding sites to a different extent. The efficiency of T7 RNAP inhibition by OA can be calculated according to the decrease in the amount of RNA synthesized in the presence of the antibiotic. Importantly, OA inhibited T7 RNAP more efficiently at CGCG (85% decrease in the full-length transcript) and GGGG (90%) sites than at GCGC (58%). Thus, OA binding to the site with the central CG dinucleotide had the weakest effect on transcription. Moreover, the addition of OA led to new RNAP stops near the positions of the binding site. These RNAP stops were apparently stronger for the sites with the central GC or GG than CG (Figure 4). The efficacy of T7 RNAP inhibition by OA correlated with the lifetime of OA complexes formed with the respective tetranucleotide sites (Figure 3, Table 2).

## 3. Discussion

Although the importance of kinetics for the mechanism of action of antibiotics had been emphasized by Donald Crothers more than five decades ago [20], the examples confirming this thesis for diverse groups of antibiotics are continuing to emerge even today. Indeed, the difference in kinetic stability of actinomycin D-DNA and actinomine-DNA complexes was critical for the activity since the short-lived actinomine–DNA complex was incapable of blocking RNAP [21]. Similarly, Behr et al. found that, among Chro derivatives, the most potent RNAP inhibitors were those that formed slowly dissociating complexes with DNA [22]. Fox et al. revealed that, compared to other anthracycline antibiotics, a high antibacterial activity of nogalamycin was associated with a considerably slower dissociation from DNA [23]. More recently, Mankin and co-authors discovered that differential dissociation kinetics of macrolide antibiotics from the ribosome correlated with their ability to cause cell death. While bacteriostatic and bactericidal macrolides bound the ribosome with comparable affinities, the bactericidal antibiotics dissociated from the ribosome with significantly slower rates [24]. In the present study, we demonstrate that antibiotic OA binds to DNA sequences of any G/C context with similar affinities and presumably form a similar type of complex. However, kinetic studies revealed slower dissociation rates of OA from SGCS and SGGS sites than from SCGS sites. This kinetic effect is manifested in transcriptional inhibition, a process most relevant to the mechanism of OA cytotoxicity. In spite of similar equilibrium constants of OA binding to SGCS, SGGS and SCGS sites, the more efficient OA inhibition of RNAP at SGCS and SGGS sites originates from the slower dissociation kinetics of OA from these sites.

An important methodological aspect of this work uncovers the limitation of EMSA and helps in interpreting its results. EMSA is a powerful yet simple technique for studies of affinity, specificity or kinetics of interaction of nucleic acids with proteins or other nucleic acids [25]. The method has versatile applications in the screening of targets, development of aptamers, etc. However, the applicability of EMSA to studies of small molecular weight ligands (e.g., antibiotics or peptides) is extremely limited, even for high affinity binders. Normally, even if the charge and the size of a small ligand are sufficient for a tangible retardation of the complex, the complex is rarely observed in the gel. The reason should be sought in the kinetics of the complex dissociation. As demonstrated earlier, the dissociation rate of large protein molecules from a nucleic acid is considerably slowed down in PAAG compared to solution [26]. However, this may not be true for smaller ligands, in which the mobility shift is undetectable due to dissociation of the complex during electrophoresis. Our measurements of OA dissociation rate from different DNA binding sites by two techniques (in solution and in the gel) provided comparable lifetimes of the complex. This implies that complexes of small molecular weight ligands with the nucleic acid are not “kinetically stabilized” by PAAG, as is the case for large proteins. In addition, our results argue against the role of electric fields in the decomposition of drug–DNA complexes in EMSA. Given the considerable difference in mobility of negatively charged DNA hairpins and a close-to-neutral OA molecule [27], the electric field may contribute to disintegration of the OA–DNA complex. However, the strength of the macroscopic electric field used in electrophoresis is several orders of magnitude lower than the forces within molecular complexes. Therefore, the external field exerts no effect on complex disintegration. This rationale is confirmed by the similar dissociation rate of OA–DNA complexes observed in EMSA and in the experiments on the passive diffusion of OA–DNA complexes in PAAG.

**Figure 5:**
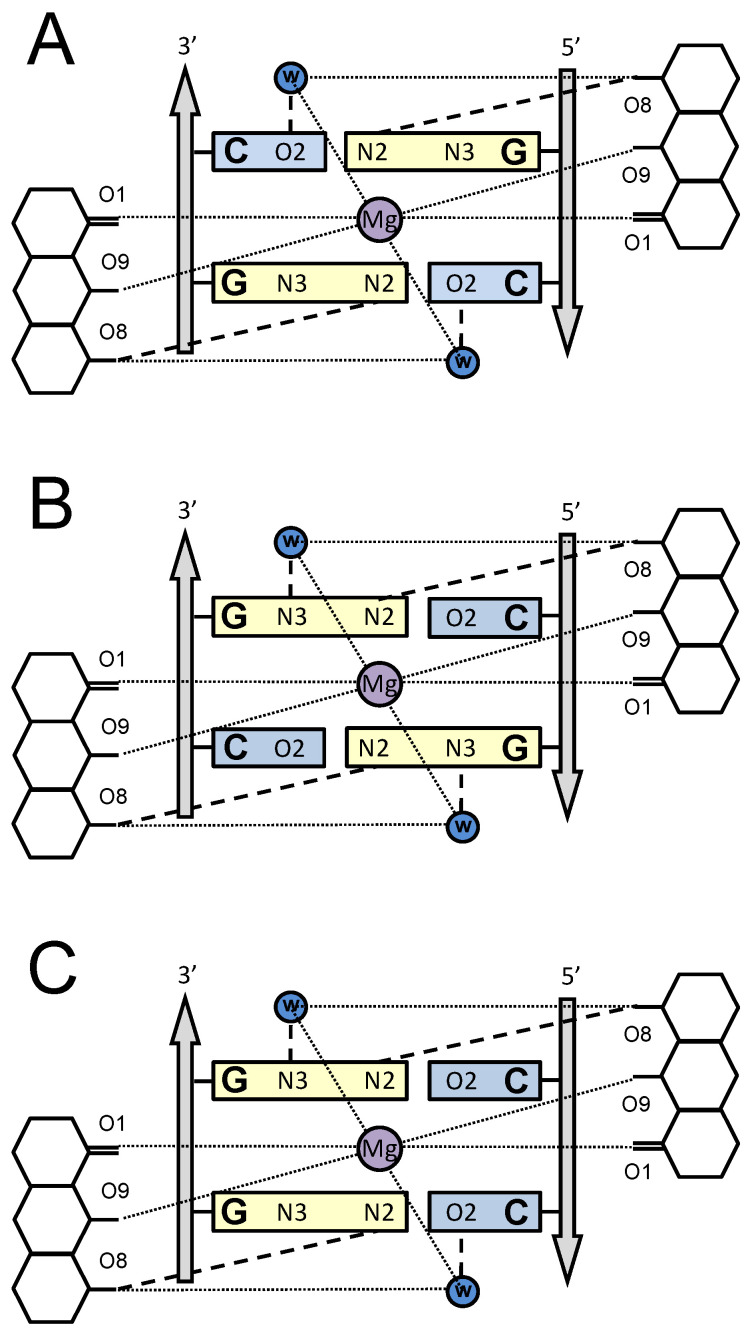
Schematic interaction of the OA aglycon and the central dinucleotide GC (**A**), CG (**B**) and GG (C) of the DNA binding site. (**A**) The network of hydrogen bonds between the minor groove edges of G-C base pairs and OA is depicted according to the X-ray structure (PDB ID: 1VAQ) of the Chro complex with self-complementary oligonucleotide 5’-TTGGCCAA containing central GC [12]. (**B**,**C**) The proposed models of hydrogen bonding in complexes of OA with binding sites containing central dinucleotides 5’-CG and 5’-GG.

An understanding of the differential behavior of OA upon interaction with three types of DNA binding sites, SGCS, SCGS or SGGS, requires consideration of molecular models of the corresponding drug–DNA complexes. High structural similarity between OA and Chro molecules (Figure 1A), as well as similar results for the two drugs in biochemical tests including footprinting assays [11,28], suggests that the pattern of interaction of OA and Chro with DNA is very much akin. The X-ray structure for the complex of Chro with GGCC binding site [12], containing the central dinucleotide GC, provided the basis for building a model of the drug interaction with the other sites containing central GG and CG dinucleotides (Figure 5). The X-ray model revealed the interaction of the minor groove edges of the two central GC base pairs with the aglycon moieties of the OA dimer and absolute requirement the amino group in guanine and the keto group in cytosine for the specific recognition and accommodation of the antibiotic’s dimer in the DNA minor groove (Figure 5A) [12]. Since the specificity of OA is defined by the two central base pairs, the models are illustrated by the scheme of interaction between the central dinucleotide, GC, CG or GG, of the binding site and the aglycon moiety of the drug. The aglycon moieties of the OA dimer interact with the G-C edges of the minor groove via direct hydrogen bonding and indirect contact via water molecules. Other types of contact between the antibiotic and DNA, such as interactions with phosphate groups, are absent in the scheme in Figure 5 since they are not specific to the nucleotide context of the binding site. The geometry of the G-C base pair within the DNA double helix is such that the substitution of a G-C base pair with a C-G would leave the position of the guanine amino group unchanged (because it is located right in the center of the minor group), while O2 of C would be replaced by N3 of G and vice versa. Since both groups are known to be efficient acceptors of H bonds, one may suppose that G-C to C-G flipping would not change the ability of the minor groove to accommodate the OA dimer (Figure 5B,C). The model of OA interaction with the three types of binding sites, SGCS, SCGS or SGGS, preserves all hydrogen bonds between the OA dimer and DNA. It is also in agreement with CD studies that argue for structural similarities of OA complexes with SGGS, SGCS or SCGS sites (Figure 2). Similar energetic parameters of the drug–DNA interaction with different sites obtained by ITC (Table 1) support this model of a similar hydrogen bond network for three types of binding sites. Contrary to the ITC results for another antibiotic, Mithramycin, that supported entropically driven binding to DNA [29], we found the enthalpy driving nature of OA’s interaction with DNA. Considerable enthalpy change upon binding is typical for most minor-groove DNA ligands due to the dominating role of hydrogen bonds in the formation of the drug–DNA complex. Although the number of hydrogen bonds is proposed to be conserved for different DNA binding sites, differences in enthalpy could be explained by less favorable H-bond angle formation for guanine N2 in the central G-C base pairs [14].

What is the structural basis for the higher dissociation rate of OA from SCGS sites compared to SGCS and SGGS? Since the hydrogen bond network is similar in OA complexes with all three binding sites, the difference must be sought in the structural properties of the double helix formed by various G/C sequences. We suggest that the differential stacking interaction between central base pairs of the binding site may influence local structural properties of DNA, thereby governing the mode of its interaction with OA. Indeed, detailed studies of self-complementary G4C4 and C4G4 oligonucleotides (differing only in the type of central stacking) revealed a distorted base stacking at the CG step in C4G4 [30,31]. In addition, the stacking energies of dinucleotides decreased in the order GC > GG > CG (2.7 > 1.97 > 1.44 kcal) [32,33]. The stacking interaction influences the local stiffness of the double helix and determines its liability for conformational rearrangements during ligand binding and dissociation. It is reasonable to assume that OA binding is accompanied by considerable conformational changes in the double helix. This assumption is supported by a seriously distorted DNA structure in the complexes with Chro, as determined by X-ray [12] and NMR [13] analyses and by footprints of the antibiotic with DNase and Fe-EDTA [11,34]. Thus, if ligand release is associated with a certain conformational DNA rearrangement, the more stable stacking would determine a higher energy barrier for such a transition, which results in a retarded OA dissociation from SGCS and SGGS sites.

## 4. Materials and Methods

### 4.1. Chemicals and DNA Oligonucleotides

All reagents were purchased from Sigma-Aldrich (St. Louis, MO, USA), unless specified otherwise. OA was produced at Gause Institute of New Antibiotics (Moscow, Russia) as described [35]. Oligodeoxynucleotides were synthesized by Evrogen JSC (Moscow, Russia). The concentration of each oligonucleotide was determined by absorption at 260 nm in water, at 90 °C, using Jasco V-550 spectrophotometer (Jasco, Tokyo, Japan). The calculated extinction coefficients were used to quantitate the concentrations.

### 4.2. Electrophoretic Mobility Shift Assays (EMSA)

Non-labeled DNA hairpins at low (50 nM) or high (2.5 µM) concentrations (indicated in figure legends) were pre-incubated for 30 min with OA in buffer A containing 100 mM NaCl, 5 mM MgCl_2_ and 20 mM Tris pH 8.0 before loading on a 10% PAAG. Electrophoresis was run for 1–2 h at 15 V/cm, either in a 45 mM Tris base/45 mM boric acid/1 mM EDTA (0.5× TBE buffer) pH 8.3 or in 0.5× TBM buffer pH 8.3 which contained 1 mM magnesium acetate instead of EDTA. The OA–DNA complexes at the high concentration were directly visualized by intrinsic fluorescence on a ChemiDoc™ XRS+ System (BioRad, Hercules, CA, USA) after UV excitation of the bound drug. Then, the same gels were stained with ethidium bromide (EtBr) for 15 min to detect DNA. The OA–DNA complexes formed at the low DNA concentration were visualized by staining the gel with SybrGold and scanning in the Typhoon FLA 9500 fluorescence scanner (GE Healthcare, Chicago, IL, USA) equipped with a 473-nm blue LD laser and LBP filter.

### 4.3. Fluorescence

Binding was performed in 96-well plates by mixing OA (5 µM) with increasing concentrations of a DNA hairpin in buffer A. The fluorescence intensity of samples was recorded at 550 nm (band width 10 nm) upon excitation at 440 nm (band width 10 nm), after a 20-min incubation at 25 °C using a fluorescence Tecan Spark^®^ plate reader (Tecan Trading AG, Zurich, Switzerland).

### 4.4. CD Spectroscopy

CD of a DNA hairpin (2.5 µM) was recorded in buffer A at 25 °C on a Jasco-j715 CD spectrometer (Jasco, Tokyo, Japan), using a quartz cell with a 10-mm optical path length after standard renaturation procedure. Monitoring of OA–DNA binding to the DNA hairpin was performed by titration of OA (5 µM) with the hairpin in buffer A at 25 °C and recording UV-Vis CD spectra. Spectra were obtained at a 1-nm bandwidth. Three scans were averaged.

### 4.5. ITC

Thermodynamic parameters of OA binding to the DNA oligonucleotide were measured using an iTC200 instrument (MicroCal, Northampton, MA, USA). Experiments were carried out at 25 °C in buffer A. Aliquots of 100 µM oligonucleotide solution in buffer A were injected into a 200-µL calorimetric cuvette containing 30 µM of OA solution in buffer A to achieve the complete binding isotherm. The heat of dilution was measured by injecting the solution of the oligonucleotide into the same buffer. The values were subtracted from the heat of the binding reaction to obtain the effective heat of binding. The resulting titration curves were fitted to a two-binding-mode model using MicroCal Origin software to determine the association constant (K), enthalpy change (ΔH) and stoichiometry (N). The entropy variation (ΔS) was calculated according to the standard thermodynamic equation. Three independent experiments were averaged.

### 4.6. Kinetics of OA-DNA Complex Formation and Dissociation

Measurements of OA–DNA binding kinetics were performed on a Cary Eclipse spectrofluorometer (Varian, Sydney, Australia) by monitoring the fluorescence intensity at 550 nm upon excitation at 440 nm. One µl of oligonucleotide (final concentration C_0_ = 2 µM) was added to OA solution (5 µM) in buffer A. Recording of fluorescence started after 10–15 s of intensive mixing and continued for 10 min. Time dependence of fluorescence intensity reflected the concentration of bound to OA DNA (C_b_) and can be approximated by a second-order kinetics equation:

C_b_(t) = С_0_ × (F(t) − F_0_)/(F_max_ − F_0_) =С_0_ × ( 1 − 1/(1 + С_0_ × k^+^ × t) (1)

Dissociation of drug–duplex complexes was initiated by adding 1% sodium dodecyl sulfate (SDS) to OA–DNA complexes (pre-incubated for 30 min in 200 µL of buffer A) and rapid mixing [36,37]. The concentration of the bound DNA was determined from fluorescence intensity and time dependence (approximated with the equation for a first-order kinetics reaction):

C_b_(t) = С_0_ × (F(t) − F_0_)/(F_max_ − F_0_) = С_0_ × exp(−k⁻ × t) (2)

The lifetime of the complex was estimated as reciprocal of k^−^.

### 4.7. Diffusion in PAAG

An alternative approach to measuring the dissociation rate of OA–DNA complexes was based on differential rates of passive diffusion of the drug and a bulkier DNA hairpin into PAAG. OA and the DNA hairpin (mixed at molar ratio 2:1) were incubated in buffer A for 30 min before adding 1/6 volume of the loading dye (50% glycerol, 0.01% bromophenol blue, 0.01% xylene cyanol). The cylindrically shaped loading wells (diameter 3 mm, depth 8 mm) were pre-formed in 10% PAAG (containing 0.5× TBE) in a Petri dish. The wells were filled with OA–DNA complexes. OA fluorescence was recorded on a ChemiDoc™ XRS+ System (BioRad, Hercules, CA, USA) under the trans-UV mode at indicated time intervals.

### 4.8. T7 RNA Polymerase Mediated Transcription

Double-stranded templates for in vitro transcription were obtained by slow renaturation of T7GC, T7GG and T7CG oligonucleotides and their complementary strands (Table S1). The reaction mixture contained 40 mM Tris-HCl (pH 7.9), 12 mM MgCl_2_, 10 mM dithiothreitol, 10 mM NaCl, 2 mM spermidine, 2 mM each dNTP, 100 nM of a double-stranded DNA template, 0.5 µM OA (where indicated) and 30 U of T7 RNA polymerase (Thermo Fisher Scientific, Waltham, MA, USA). After 1 h of incubation at 37 °C, the reaction was stopped by the addition of EDTA to 20 mM, 1/3 volume of the loading buffer (90% formamide, 0.025% bromophenol blue, 0.025% xylene cyanol) and the solution was heated for 10 min at 70 °C. An aliquot was loaded onto a 10% denaturing polyacrylamide gel. Gels were stained with SYBR Gold (Thermo Fisher Scientific, Waltham, MA, USA) and visualized on a Typhoon FLA 9500 fluorescence scanner (GE Healthcare, Chicago, IL, USA).

## 5. Conclusions

In this work, we have shown that antibiotic olivomycin A, which targets the minor groove of the G/C rich double-stranded DNA, has in fact a narrower specificity. Although the antibiotic binds diverse G/C DNA sequences with similar affinity, it is able to differentiate between them on the basis of the considerably different lifetimes of the complex. In particular, central dinucleotide CG in the binding site forms a kinetically less stable complex with olivomycin A dimer compared to dinucleotides GG or GC. We have demonstrated that this dissociation kinetics of the drug–DNA complex may be crucial for the activity of the antibiotic via the mechanism of transcription inhibition.

## Figures and Tables

**Table 1:** Thermodynamic parameters of OA binding to G/C sites obtained by isothermal titration calorimetry (ITC) at 25 °C. Oligo numbering is according to Figure 1B.

Oligo Number	N (DNA/OA Dimer)	K_a_, 10^7^ (M^−1^)	ΔH (kcal/mol)	TΔS (kcal/mol)
1	0.9	2.08 ± 0.24	−6.97 ± 0.05	3.0
2	1.0	1.88 ± 0.15	−7.60 ± 0.04	2.3
3	0.7	2.3 ± 0.7	−9.68 ± 0.18	0.4
4	1.0	1.94 ± 0.22	−6.50 ± 0.05	3.5
5	1.0	2.21 ± 0.18	−7.39 ± 0.04	2.6
6	1.1	1.8 ± 0.3	−7.18 ± 0.08	2.7
7	0.9	1.9 ± 0.3	−5.11 ± 0.06	4.8
8	0.9	1.26 ± 0.15	−4.42 ± 0.04	5.3
9	0.9	1.16 ± 0.17	−4.76 ± 0.05	4.9
10	0.9	1.11 ± 0.10	−5.39 ± 0.03	4.2

**Table 2:** Parameters of OA binding and dissociation for ten G/C sites.

Central Dinucleotide	Binding Site (Oligo Number)	*k^+^*, 10^4^ M^−1^ s^−1^	*k^−^*_(SDS)_, 10^−4^ s^−1^	Lifetime of OA–DNA Complexes, Min *
GG	GGGG (1)	3.8 ± 0.4	1.3 ± 0.2	128
	GGGC (2)	4.5 ± 0.2	2.5 ± 0.1	66
	CGGG (3)	3.9 ± 0.3	1.4 ± 0.4	122
	CGGC (4)	3.4 ± 0.2	3.8 ± 0.2	44
GC	GGCG (5)	3.0 ± 0.2	1.3 ± 0.1	129
	GGCC (6)	2.7 ± 0.3	1.7 ± 0.4	99
	CGCG (7)	1.8 ± 0.3	1.4 ± 0.1	118
CG	GCGG (8)	3.6 ± 0.1	16 ± 1	10
	GCGC (9)	3.5 ± 0.4	17 ± 1	10
	CCGG (10)	4.7 ± 0.2	10 ± 1	17

* Calculated as reciprocal of *k*^−^_(SDS)_.

## Supplementary Materials

Supplementary Materials can be found at https://www.mdpi.com/1422-0067/21/15/5299/s1.

## References

[1] Lombo F., Menendez N., Salas J.A., Mendez C. (2006). The aureolic acid family of antitumor compounds: Structure, mode of action, biosynthesis, and novel derivatives. Appl. Microbiol. Biotechnol..

[2] Chakraborty H., Devi P.G., Sarkar M., Dasgupta D. (2008). Multiple functions of generic drugs: Future perspectives of aureolic acid group of anti-cancer antibiotics and non-steroidal anti-inflammatory drugs. Mini Rev. Med. Chem..

[3] Hu L., Chen Q., Wang Y., Zhang N., Meng P., Liu T., Bu Y. (2019). Sp1 Mediates the Constitutive Expression and Repression of the PDSS2 Gene in Lung Cancer Cells. Genes.

[4] Mitra P., Eckenrode J.M., Mandal A., Jha A.K., Salem S.M., Leggas M., Rohr J. (2018). Development of Mithramycin Analogues with Increased Selectivity toward ETS Transcription Factor Expressing Cancers. J. Med. Chem..

[5] Gomez K., Sandoval A., Barragan-Iglesias P., Granados-Soto V., Delgado-Lezama R., Felix R., Gonzalez-Ramirez R. (2019). Transcription Factor Sp1 Regulates the Expression of Calcium Channel alpha2delta-1 Subunit in Neuropathic Pain. Neuroscience.

[6] Ming L.J. (2003). Structure and function of “metalloantibiotics”. Med. Res. Rev..

[7] Simonova V.S., Samusenko A.V., Filippova N.A., Tevyashova A.N., Lyniv L.S., Kulik G.I., Chekhun V.F., Shtil A.A. (2005). Olivomycin induces tumor cell apoptosis and suppresses p53-induced transcription. Bull. Exp. Biol. Med..

[8] Sergeev A.V., Tevyashova A.N., Vorobyov A.P., Gromova E.S. (2019). The Effect of Antitumor Antibiotic Olivomycin A and Its New Semi-synthetic Derivative Olivamide on the Activity of Murine DNA Methyltransferase Dnmt3a. Biochem. Biokhimiia.

[9] Aich P., Sen R., Dasgupta D. (1992). Role of magnesium ion in the interaction between chromomycin A3 and DNA: Binding of chromomycin A3-Mg^2+^ complexes with DNA. Biochemistry.

[10] Chakrabarti S., Bhattacharyya D., Dasgupta D. (2000). Structural basis of DNA recognition by anticancer antibiotics, chromomycin A(3), and mithramycin: Roles of minor groove width and ligand flexibility. Biopolymers.

[11] Fox K.R., Howarth N.R. (1985). Investigations into the sequence-selective binding of mithramycin and related ligands to DNA. Nucleic Acids Res..

[12] Hou M.H., Robinson H., Gao Y.G., Wang A.H. (2004). Crystal structure of the [Mg^2+^-(chromomycin A3)2]-d(TTGGCCAA)2 complex reveals GGCC binding specificity of the drug dimer chelated by a metal ion. Nucleic Acids Res..

[13] Gao X.L., Mirau P., Patel D.J. (1992). Structure refinement of the chromomycin dimer-DNA oligomer complex in solution. J. Mol. Biol..

[14] Hou M.H., Lu W.J., Lin H.Y., Yuann J.M. (2008). Studies of sequence-specific DNA binding, DNA cleavage, and topoisomerase I inhibition by the dimeric chromomycin A3 complexed with Fe(II). Biochemistry.

[15] Liu C., Chen F.M. (1994). Oligonucleotide studies of sequence-specific binding of chromomycin A3 to DNA. Biochemistry.

[16] Carpenter M.L., Marks J.N., Fox K.R. (1993). DNA-sequence binding preference of the GC-selective ligand mithramycin. Deoxyribonuclease-I/deoxyribonuclease-II and hydroxy-radical footprinting at CCCG, CCGC, CGGC, GCCC and GGGG flanked by (AT)n and An.Tn. Eur. J. Biochem..

[17] Hampshire A.J., Fox K.R. (2008). The effects of local DNA sequence on the interaction of ligands with their preferred binding sites. Biochimie.

[18] Fletcher M.C., Fox K.R. (1996). Visualising the dissociation of sequence selective ligands from individual binding sites on DNA. Febs Lett..

[19] Yoshizawa S., Kawai G., Watanabe K., Miura K., Hirao I. (1997). GNA trinucleotide loop sequences producing extraordinarily stable DNA minihairpins. Biochemistry.

[20] Crothers D.M., Hahn F.E. (1971). Kinetics of Binding of Drugs to DNA. Proceedings of the Research Symposium on Complexes of Biologically Active Substances with Nucleic Acids and Their Modes of Action: Held at the Walter Reed Army Institute of Research Washington, 16–19 March 1970.

[21] Muller W., Crothers D.M. (1968). Studies of the binding of actinomycin and related compounds to DNA. J. Mol. Biol..

[22] Behr W., Honikel K., Hartmann G. (1969). Interaction of the RNA polymerase inhibitor chromomycin with DNA. Eur. J. Biochem..

[23] Fox K.R., Brassett C., Waring M.J. (1985). Kinetics of dissociation of nogalamycin from DNA: Comparison with other anthracycline antibiotics. Biochim. Et Biophys. Acta.

[24] Svetlov M.S., Vazquez-Laslop N., Mankin A.S. (2017). Kinetics of drug-ribosome interactions defines the cidality of macrolide antibiotics. Proc. Natl. Acad. Sci. USA.

[25] Hellman L.M., Fried M.G. (2007). Electrophoretic mobility shift assay (EMSA) for detecting protein-nucleic acid interactions. Nat. Protoc..

[26] Fried M., Crothers D.M. (1981). Equilibria and kinetics of lac repressor-operator interactions by polyacrylamide gel electrophoresis. Nucleic Acids Res..

[27] Beniaminov A.D., Dezhenkova L.G., Mamaeva O.K., Shchyolkina A.K., Tevyashova A.N., Kaluzhny D.N., Shtil A.A. (2018). Divalent cations are dispensable for binding to DNA of a novel positively charged olivomycin A derivative. PLoS ONE.

[28] Van Dyke M.W., Dervan P.B. (1983). Chromomycin, mithramycin, and olivomycin binding sites on heterogeneous deoxyribonucleic acid. Footprinting with (methidiumpropyl-EDTA)iron(II). Biochemistry.

[29] Barcelo F., Scotta C., Ortiz-Lombardia M., Mendez C., Salas J.A., Portugal J. (2007). Entropically-driven binding of mithramycin in the minor groove of C/G-rich DNA sequences. Nucleic Acids Res..

[30] Trantirek L., Stefl R., Vorlickova M., Koca J., Sklenar V., Kypr J. (2000). An A-type double helix of DNA having B-type puckering of the deoxyribose rings. J. Mol. Biol..

[31] Stefl R., Trantirek L., Vorlickova M., Koca J., Sklenar V., Kypr J. (2001). A-like guanine-guanine stacking in the aqueous DNA duplex of d(GGGGCCCC). J. Mol. Biol..

[32] Protozanova E., Yakovchuk P., Frank-Kamenetskii M.D. (2004). Stacked-unstacked equilibrium at the nick site of DNA. J. Mol. Biol..

[33] Vologodskii A., Frank-Kamenetskii M.D. (2018). DNA melting and energetics of the double helix. Phys. Life Rev..

[34] Cons B.M., Fox K.R. (1990). The GC-selective ligand mithramycin alters the structure of (AT)n sequences flanking its binding sites. FEBS Lett..

[35] Gause G.F., Sartorelli A.C., Johns D.G. (1975). Chromomycin, Olivomycin, Mithramycin. Antineoplastic and Immunosuppressive Agents: Part II.

[36] Chen F.M. (1997). Methods for the studies of drug dissociation from DNA. Methods Mol. Biol..

[37] Phillips D.R., Crothers D.M. (1986). Kinetics and sequence specificity of drug-DNA interactions: An in vitro transcription assay. Biochemistry.

