# Supplementary material for: Discrimination between G/C Binding Sites by Olivomycin A Is Determined by Kinetics of the Drug-DNA Interaction
Source: Int J Mol Sci. 2020 Jul 26;21(15):5299. doi: 10.3390/ijms21155299 (PMC7432603; doi:10.3390/ijms21155299)

Supplementary Material

Discrimination between G/C binding sites by Olivomycin A is determined by kinetics of the drug-DNA interaction

Artemy D. Beniaminov[1,*], Galina V. Chashchina[1], Mikhail A. Livshits[1], Olga I. Kechko[1], Vladimir A Mitkevich[1], Olga K. Mamaeva[1], Anna N. Tevyashova[2], Alexander A. Shtil[3,4], Anna K. Shchyolkina[1], Dmitry N. Kaluzhny[1,*]

[1]Engelhardt Institute of Molecular Biology, Russian Academy of Sciences, 32 Vavilov Street, 119991 Moscow, Russia

[2]Gause Institute of New Antibiotics, 11 B. Pirogovskaya Street, 119021 Moscow, Russia

[3]Blokhin National Medical Research Center of Oncology, 24 Kashirskoye shosse, 115478 Moscow, Russia

[4]Institute of Gene Biology, Russian Academy of Sciences, 34/5 Vavilov Street, 119991 Moscow, Russia

*To whom correspondence should be addressed.

e-mail: abeniaminov@mail.ru. Correspondence may also be addressed to Dmitry Kaluzhny.

E-mail: uzhny@mail.ru

Table S1. Oligodeoxynucleotides used in the study 2

Figure S1. ITC raw data……………………………………………………………………………..3

Figure S2. Vis CD spectra……………………………………………………………………………..6

Figure S3. UV CD spectra……………………………………………………………………………..7

Figure S4. UV-VIS CD Spectra of the OA-DNA complexes containing binging…………………….…...8

Figure S5. EMSA ………………………………………………………………………………………9

Figure S6. Kinetics of OA binding to DNA hairpins 1-10 carrying the tetranucleotide binding site….....10

Table S1. Oligodeoxynucleotides used in the study.

Oligonucleotide name	Oligonucleotide sequence*
(1) G**GG**G	GTATTG**GG**GTTAGAATAAC**CC**CAATAC
(2) G**GG**C	GTATTG**GG**CTTAGAATAAG**CC**CAATAC
(3) C**GG**G	GTATTC**GG**GTTAGAATAAC**CC**GAATAC
(4) C**GG**C	GTATTC**GG**CTTAGAATAAG**CC**GAATAC
(5) G**GC**G	GTATTG**GC**GTTAGAATAAC**GC**CAATAC
(6) G**GC**C	GTATTG**GC**CTTAGAATAAG**GC**CAATAC
(7) C**GC**G	GTATTC**GC**GTTAGAATAAC**GC**GAATAC
(8) G**CG**G	GTATTG**CG**GTTAGAATAAC**CG**CAATAC
(9) G**CG**C	GTATTG**CG**CTTAGAATAAG**CG**CAATAC
(10) C**CG**G	GTATTC**CG**GTTAGAATAAC**CG**GAATAC
T7**GG**	**TAATACGACTCACTATA**GGACTCAGTCATGTATC**CC**CTATGTACTTTC
T7**GG**-comp **	GAAAGTACATAG**GG**GATACATGACTGAGTCCTATAGTGAGTCGTATTA
T7**GC**	**TAATACGACTCACTATA**GGACTCAGTCATGTATC**GC**GTATGTACTTTC
T7**GC**-comp	GAAAGTACATAC**GC**GATACATGACTGAGTCCTATAGTGAGTCGTATTA
T7**CG**	**TAATACGACTCACTATA**GGACTCAGTCATGTATG**CG**CTATGTACTTTC
T7**CG**-comp	GAAAGTACATAG**CG**CATACATGACTGAGTCCTATAGTGAGTCGTATTA

* Binding sites with nucleotide sequences that fit to a common pattern **SGGS** are colored in green; **SGCS** are in blue; **SCGS** are in red (S stands for G or C)

** Comp, complementary strand.

Figure S1. ITC raw data

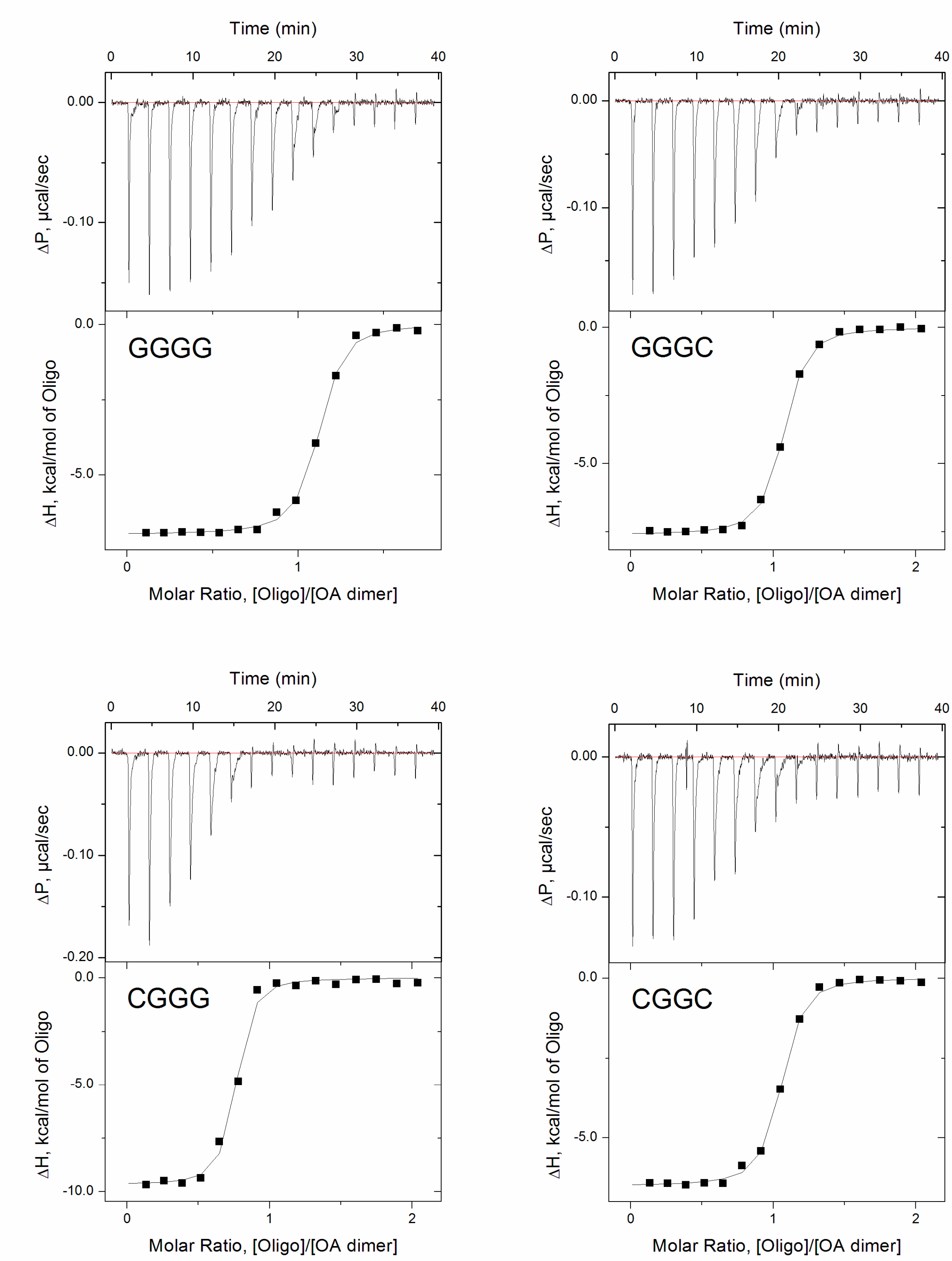

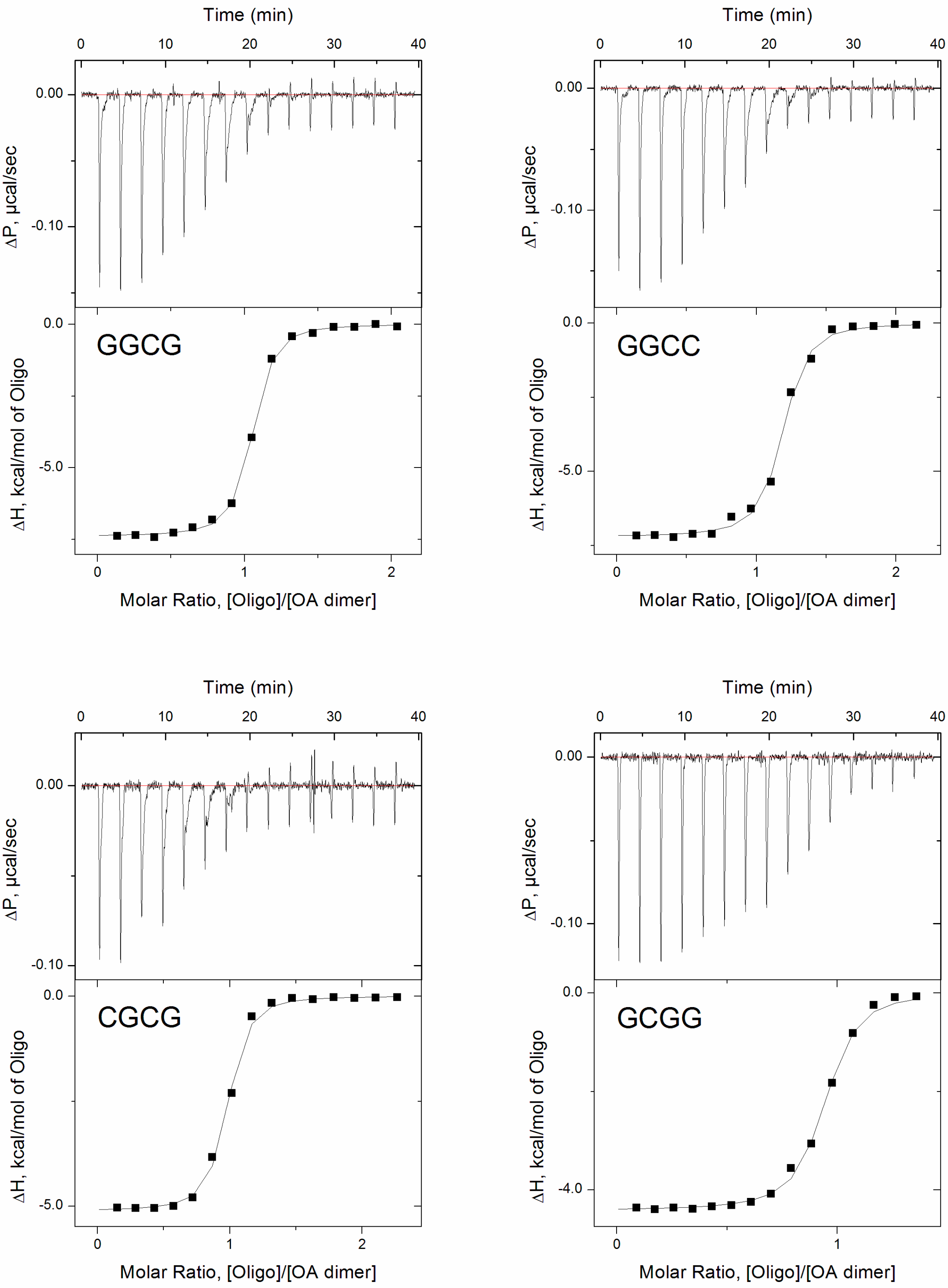
Time (min)
0
10
20
30
40
0.00
-0.10
ΔP, µcal/sec
0.0
-5.0
ΔH, kcal/mol of Oligo
GGCG
GGCC
CGCG
GCGG
-4.0
0
1
2
Molar Ratio, [Oligo]/[OA dimer]

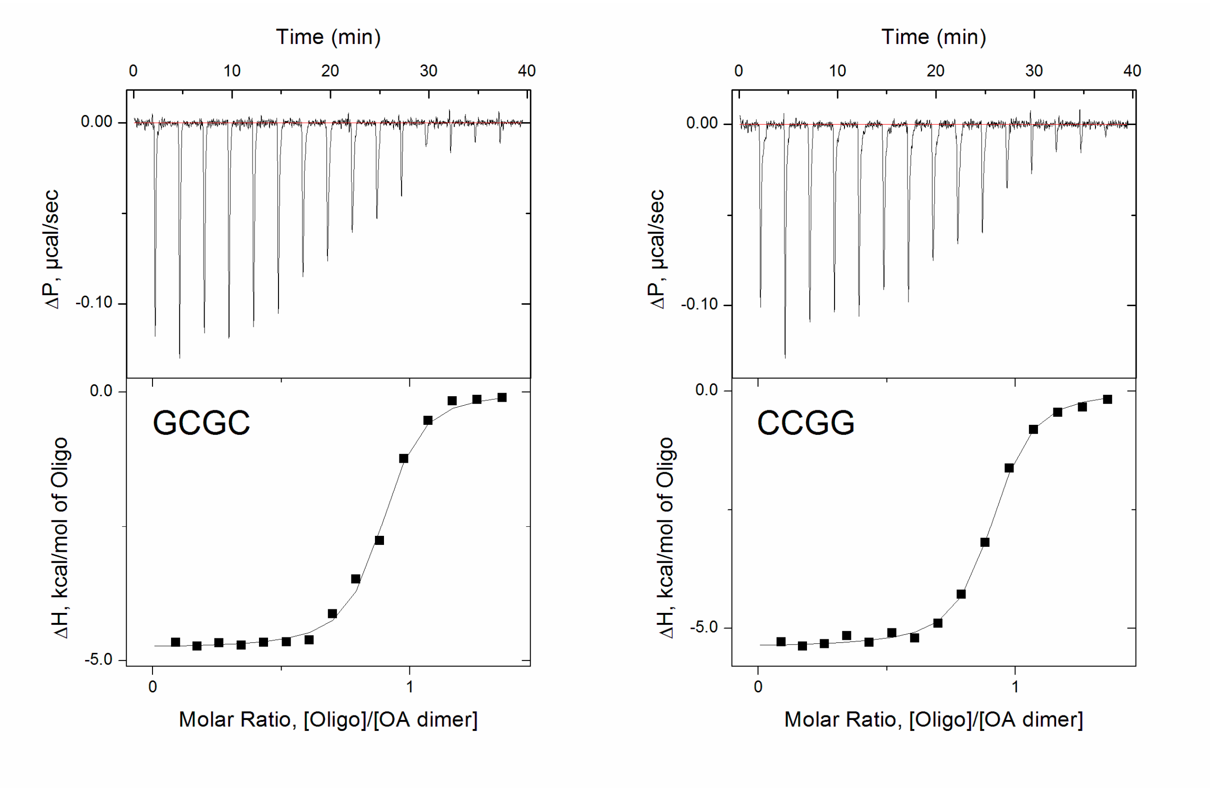
Time (min)
0
10
20
30
40
0.00
-0.10
ΔP, μcal/sec
0.0
-5.0
ΔH, kcal/mol of Oligo
GCGC
0
1
Molar Ratio, [Oligo]/[OA dimer]
Time (min)
0
10
20
30
40
0.00
-0.10
ΔP, μcal/sec
0.0
-5.0
ΔH, kcal/mol of Oligo
CCGG
0
1
Molar Ratio, [Oligo]/[OA dimer]

Figure S2. Vis CD spectra

Binding of OA to different tetranucleotide sites in solution.

CD changes at 440nm upon titration of 5 μM OA with increasing concentrations of the DNA hairpins 1, 6, 7, 9, or 10.

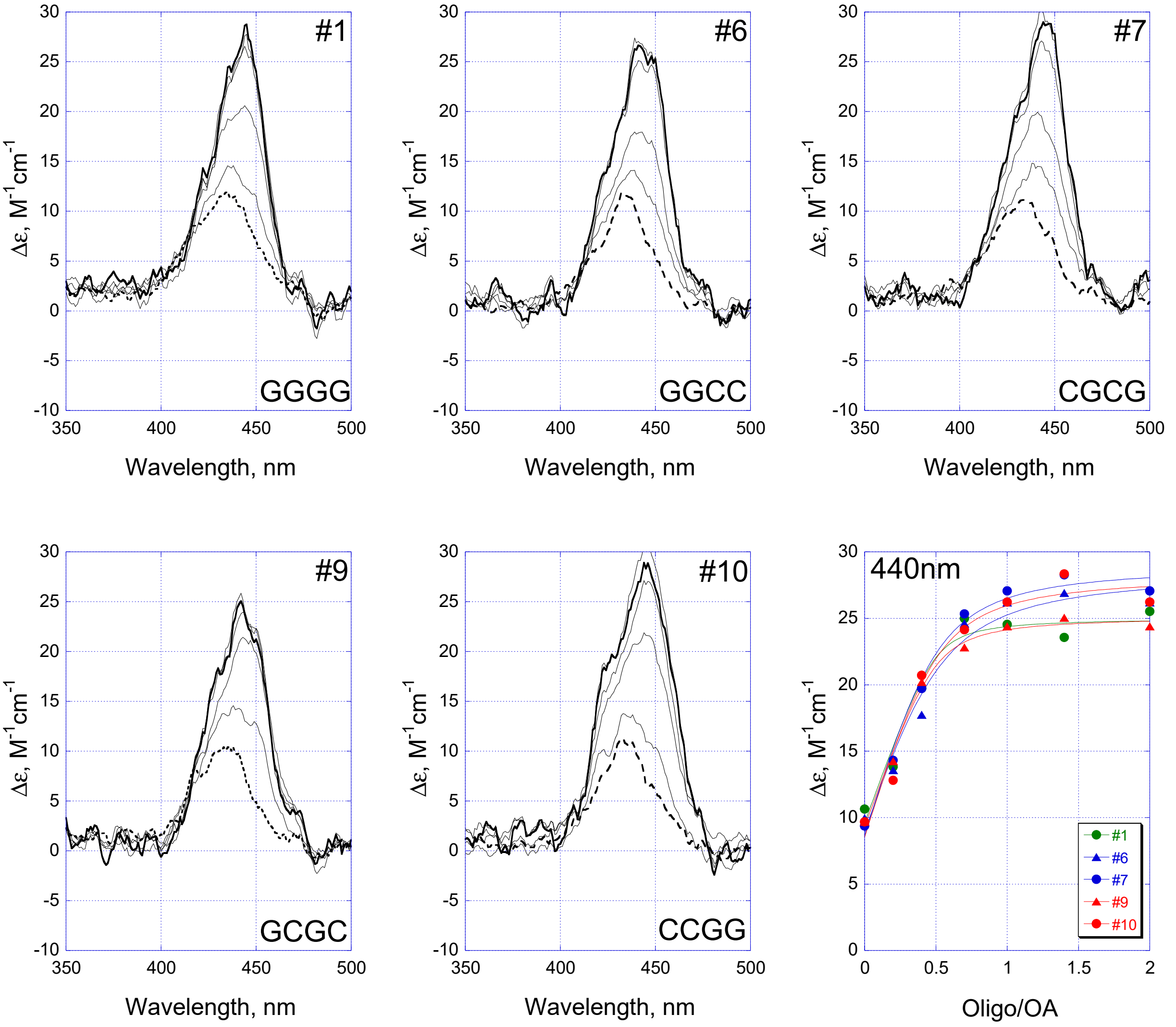

Figure S3. UV CD spectra

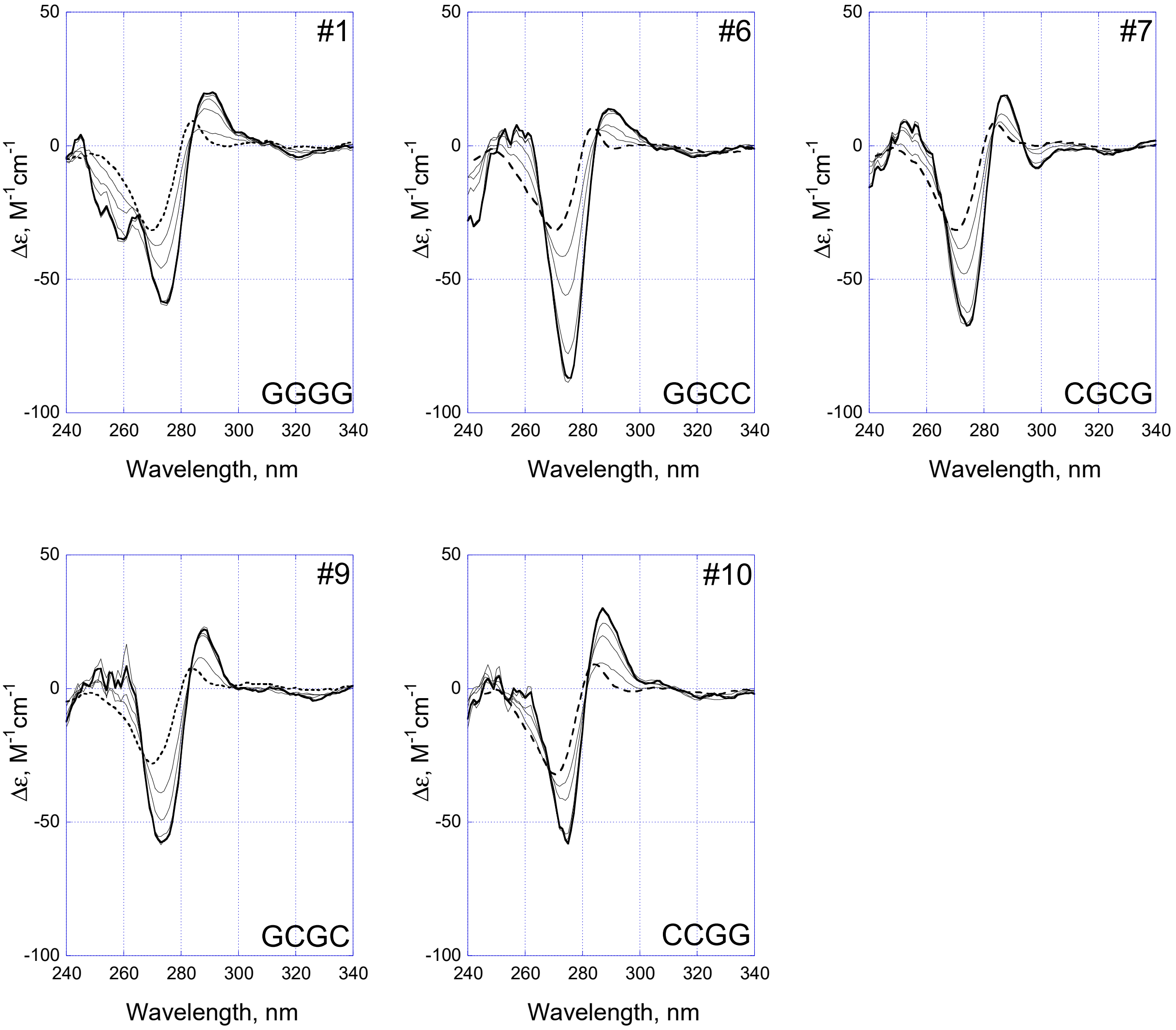

Figure S4. UV-VIS CD Spectra of the OA-DNA complexes containing binging

CD spectra of free OA (black), OA bound to hairpins 1 (binding site GGGG, green), 7 (CGCG, blue) or 9 (GCGC, red). *Inset*: CD spectra of free DNA hairpins 1, 7 and 9 (same color code).

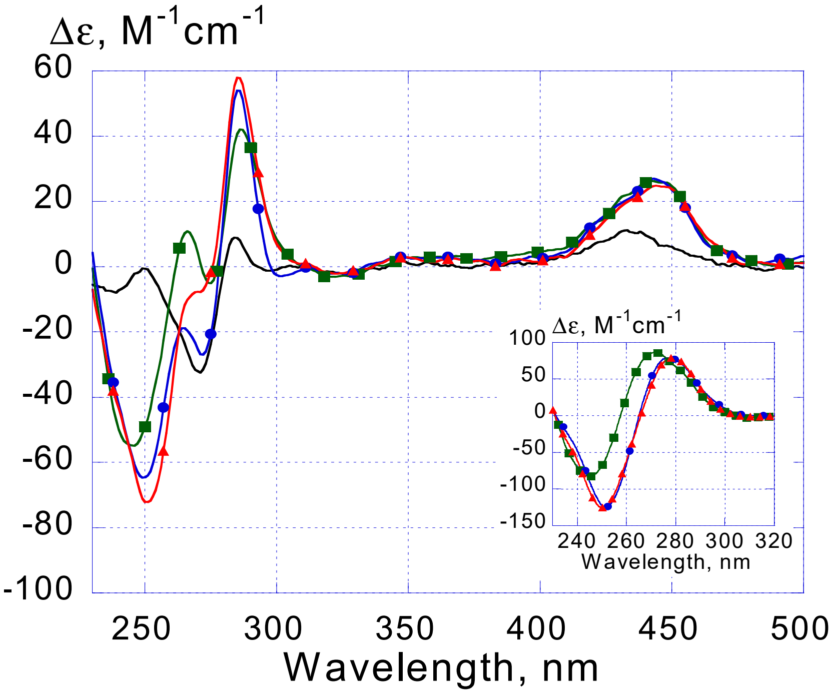

Figure S5. EMSA

(A) OA binding to DNA hairpins at high concentration: 2.5 µM OA; 5 µM DNA. The same gel was visualized upon UV excitation before (upper panel; intrinsic OA fluorescence) and after (lower panel; DNA) EtBr staining.

(B) EMSA in absence of EDTA in 0.5x TBM buffer.

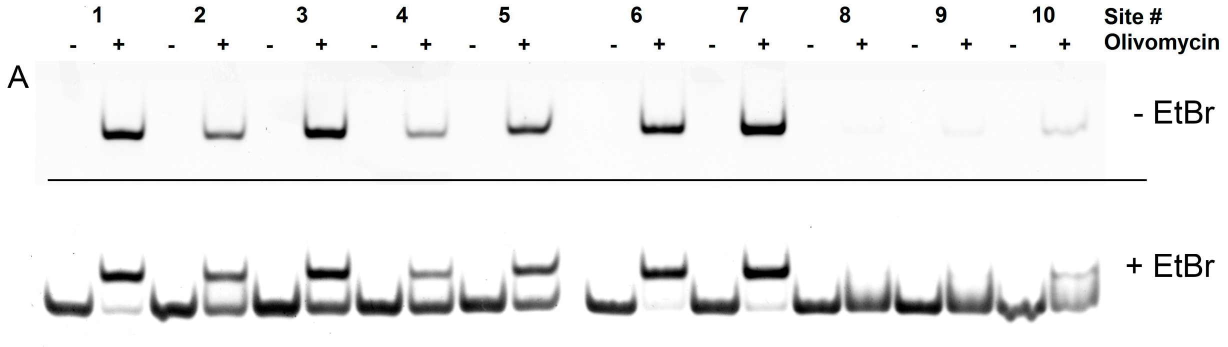

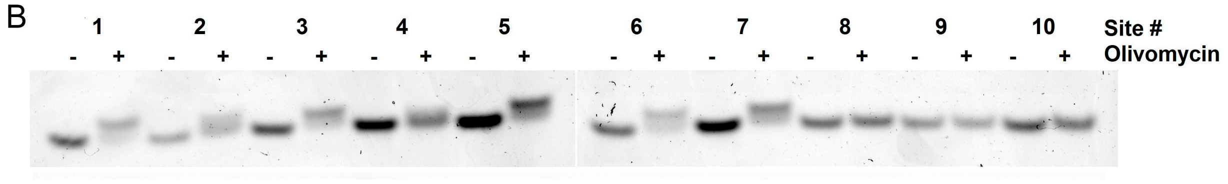

Figure S6. Kinetics of OA binding to DNA hairpins 1-10 carrying the tetranucleotide binding site. Binding sites with a common SGGS pattern are colored in green; SGCS sites are in blue; SCGS sites are in red.

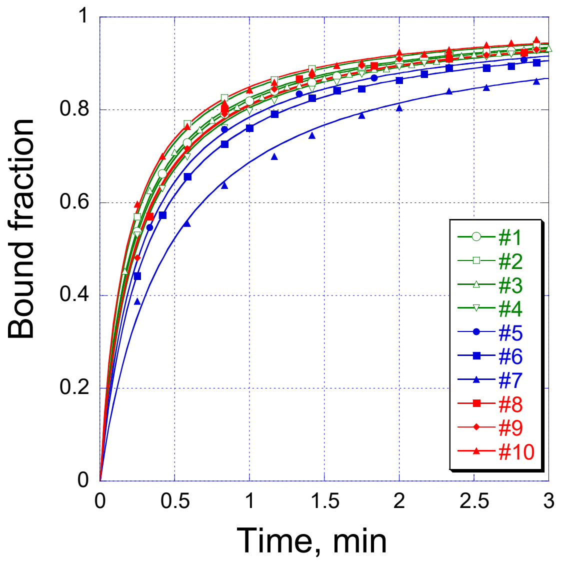

Supplement: Supplementary file 1 [file ijms-21-05299-s001.pdf]
